# Blockchains for Secure Digitized Medicine

**DOI:** 10.3390/jpm9030035

**Published:** 2019-07-13

**Authors:** Khaled Shuaib, Heba Saleous, Karim Shuaib, Nazar Zaki

**Affiliations:** 1 College of Information Technology, United Arab Emirates University, Sheikh Khalifa Bin Zayed Street, P.O. Box 15551, Al Ain, UAE; 2 Renaissance School of Medicine, Stony Brook University, Stony Brook, NY 11794, USA

**Keywords:** blockchains, healthcare, electronic medical records, privacy, security, personalized medicine

## Abstract

Blockchain as an emerging technology has been gaining in popularity, with more possible applications to utilize the technology in the near future. With the offer of a decentralized, distributed environment without the need for a third trusted party (TTP), blockchains are being used to solve issues in systems that are susceptible to cyberattacks. One possible field that could benefit from blockchains that researchers have been focusing on is healthcare. Current healthcare information systems face several challenges, such as fragmented patient data, centralized systems which are viewed as single points of attacks, and the lack of patient-oriented services. In this paper, we investigate and analyze recent literature related to the use of blockchains to tackle issues found in modern healthcare information systems. This is done to understand issues that researchers commonly focus on, to discover remaining areas of concern in any proposed solution, and to understand the possible directions of the integration of blockchains in healthcare and personalized medicine. Background information regarding blockchains and existing healthcare information systems is reviewed, followed by the methodology used in the preparation of this review, where the research questions to consider are stated. Afterwards, an analysis of the results is provided, concluding with a discussion of the remaining issues that need to be focused on, and how blockchains could benefit the healthcare sector and empower personalized medicine.

## 1. Introduction

Humans strive to remain as healthy as possible and live as full a life as possible. Therefore, healthcare is an important part of everyone’s life. In an effort to provide proper healthcare by care givers, patient records are kept at clinics and hospitals. These records help physicians understand a patient’s past diagnoses and current health status. Up until recently, medical records have been kept as physical files. Although this might not have been a major issue for hospitals or clinics, for patients, moving their medical records across caregivers, it constitutes a burden. Other issues with paper-based records have been data loss—due to various possible reasons—and the difficulty associated with data recovery. 

Electronic Health or Medical Records (EHR/EMRs) have improved healthcare infrastructure by making it easier for doctors to store, view, share, and update patient records. However, as with any electronic records system, security and privacy issues have become a challenge. One other concern is the cost of adopting the infrastructure required for keeping electronic records. Initial costs include the hardware and software needed to run an electronic healthcare system, maintenance and updates, and staff training [1,2,3]. Basic computer literacy is a requirement for users in order to be able to use the system. Otherwise, without training, hospital staff have initially found it hard to organize information, and to generate and format reports without aid [2,4]. Without an understanding of how to use the electronic healthcare system, staff may store or update records with incorrect information. This may lead to wrong diagnoses, the ordering of incorrect medical procedures, and the prescribing of the wrong medications and doses. This could result in health complications or even the death of a patient. This is the reason staff training should be included in the cost of implementing an electronic healthcare system. However, the introduction of a new system, even with training, may lead to an initial service disruption [3]. 

Another issue with EMR systems is the fragmentation of patient data. Since patients may go to different clinics, fragmented patient data may exist in different locations. Although digitizing patients’ data has eased the sharing of EMRs, an issue still lies in achieving interoperability among healthcare information systems as clinics may use different EMR systems. This implies that patients’ data can exist in different formats, which may put the patient at risk due to the time it takes to reformat the data into a readable form, possibly lose data, and the different ways that professionals can fill in information in a record [5,6,7,8]. In a risky, sensitive environment such as a hospital, one minute can be the difference between life and death for a patient. Healthcare professionals cannot afford to have their time lost due to fragmented records and interoperability issues.

Aside from fragmentation and interoperability challenges, some privacy issues can arise from the use of EMRs. This comes from healthcare infrastructures not being patient-centric; although patients own the information they provide to professionals, they do not control the EMRs themselves. This also implies that patients do not control who views their data and where it is sent or stored [8,9,10]. Some regulations were developed in order to address concerns about patient’s privacy, such as the Health Insurance Portability and Accountability Act (HIPAA) in the United States and the General Data Protection Regulation (GDPR) in Europe. Although these regulations may add some more control over a patient’s data, they do not entirely prevent the disclosure, intentional or accidental, of private data. Patients may continue to feel uneasy about their data being stored and exchanged electronically. 

The actual exchange of EMRs between two parties also needs to be secured. EMRs within an organization are typically part of a centralized healthcare infrastructure stored as part of a database. This centralized infrastructure may have a single point of attack that, if successfully brought down by cybercriminals, could hinder healthcare services. Cybercriminals can benefit from stolen EMR data either by selling it to other interested parties or using it to ask for ransoms in what has become known as “Hacking for Ransom”. Cybercriminals may also use patients’ data in an attempt to obtain prescription drugs for themselves or for others. 

In addition to stealing and misusing patients’ information, fraud also remains an issue with EHR and EMR systems. There are two possible kinds of fraud: prescription and insurance. Prescription fraud occurs when the details of a prescription are altered or duplicated to receive certain medications that cannot be obtained normally [9]. These medications may have drug-like effects, such as opioids or anti-depressants. Insurance fraud occurs when insurance companies raise the price of provided insurance while reducing the benefits, or when a medical professional enters an incorrect diagnosis for the patient in order to submit false insurance claims [11]. This not only results in a higher healthcare cost for the patient, but it also allows medical professionals to take advantage of the patient and portray false information as facts.

There have been several proposed methods of improving typical healthcare infrastructures. An example of a proposed solution is the use of cloud systems to provide a flexible, decentralized, secure service, such as the one discussed in References [12,13]. The latter, however, also incorporated blockchain technology in the proposed solution. More recently, other researchers have begun proposing the use of blockchains to improve electronic healthcare systems, especially in terms of handling EMRs. This is due to EMRs containing private information that patients would like to keep hidden from unauthorized parties. This is achievable by employing blockchains. 

### 1.1. Blockchains

**Figure 1:**
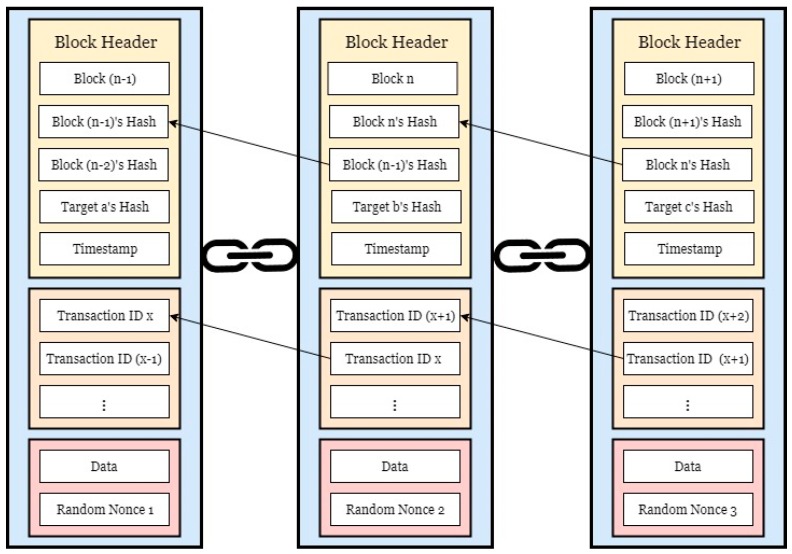
A conceptual look at a blockchain.

Blockchains were first introduced to the world by Satoshi Nakamoto in the form of the popular cryptocurrency, Bitcoin [14]. Each user in a peer-to-peer (P2P) network is considered a node, and the transactions that occur are grouped as blocks. The blocks are then connected to each other in a chain. Each node has at least one public-private key pair. The public key is used to address the node as a sender or a receiver, while the associate private key is used by a sender to sign transactions being sent and by a receiver to redeem them. [14,15,16]. In addition to requiring the correct key to decrypt and access the data, an agreement needs to be reached among the participating nodes before changes can be made [14,15,16]. This ensures that all copies of the blockchain ledger are synchronized throughout the network. Whenever changes occur on the chain, every node in the network is notified. Because of the way blocks are chained together, blockchains are immutable, private, anonymous, time-stamped ledgers. The concept shown in Figure 1 depicts how blocks are linked together in a chain and the kind of data they may contain. The block header contains data such as a block number, its hash value, the previous block’s hash, a hash of the target node, and a timestamp. Aside from the header, a list of transactions is kept. This is a major component of blockchains, as it keeps track of any and all valid transactions and requests that occur within the blockchain network. Finally, some data may be included in the block, as well as a random nonce. This randomized value is typically added to a hashed block for the purpose of “mining”. In mining, nodes known as “miners” use their computational power to solve numerical puzzles. The resulting values are used for consensus purposes to generate a valid block [16]. The random nonce sought by miners is used to generate a valid hash of the block such that a certain criteria or threshold is met once it is entered into the hashing algorithm.

The decentralized, distributed nature of blockchains also makes it more difficult to attack the network. Because a copy of the ledger exists on every node in the P2P network, transactions and data can be recoverable. If one end node is compromised or attacked, the information and connections in a blockchain network remain intact since it exists in all other nodes [17]. This also prevents unauthorized modification of data. 

There are three major types of blockchains: public, private, and consortium. [15,18]. The selection of the type of blockchain depends on the use case, since each has its own functional requirements:

▪ Public blockchains, as the name suggests, are available to the public. They are permissionless, meaning that anyone can join the blockchain network to participate in the transactions that occur [15,18]. When one node attempts to perform an action, such as modifying or adding a value, all nodes on the network are notified, as well as take part in the decision-making process. A popular example of a public blockchain is Bitcoin; anyone can join the Bitcoin network and participate in blockchain management.
▪ Private blockchains may be more beneficial for organizations This type is only available to the employees within an organization, should they choose to become a node and participate in transactions. Users outside of the organization are restricted from joining these blockchains. However, since the blockchain only exists within the organization, this kind of a blockchain system can be considered centralized [15,18].
▪ Consortium blockchains are more open than private blockchains. They can be considered as permissioned public blockchains because of their limited availability to the public. In this type of blockchain, a select group of entities participate in transaction validation and/or blockchain management. Depending on the permission rights configured by the administrations, public users may have read permissions, but may not participate in the consensus scheme used for decision making [15].

In order to reach an agreement over the next valid block of transactions, a consensus algorithm is used. Some common consensus algorithms are:

▪ Proof-of-Work (PoW): This algorithm employs a node’s Central Processing Unit (CPU) power to compete with other nodes in solving a hashing puzzle to retrieve a predetermined value [14,17]. Succeeding in doing this rewards the node with consensus power, which is determined by a certain amount of newly gained cryptocurrency. Participating nodes, or miners, can do this alone or team up with other nodes. However, due to the amount of computational power required to succeed, PoW can be computationally expensive and high energy consuming.
▪ Proof-of-Stake (PoS): The difficulty of the hashing problems solved to calculate a predetermined value is based on the assets owned by the node [15,17,19]. Miners with more assets will be more likely to create new blocks to add to the chain. This consensus method uses less computational power than PoW but may be unfair because nodes with more assets will dominate more. This may motivate poorer nodes to attempt to attack the network.
▪ Practical Byzantine Fault Tolerance (PBFT): This type of consensus algorithm involves settling disputes between nodes in a network, typically in consortium blockchains. The main goal of PBFT is to solve the Byzantine Generals Problem, where it is possible that some nodes in a network may be corrupt and attempt to send the wrong message. There is the assumption that no more than 1/3 of the total number of nodes in the network are faulty [15,20]. The node selected for the transaction needs to receive a vote from 2/3 of the other nodes before being able to continue with the transaction and add a block to the chain [15,20].

Proof of X (PoX) consensus algorithms generally depend on a qualification to decide which node has the ability to generate a new block to add to the chain [21]. PoX algorithms are found to be used for public blockchains, where nodes are required to prove that they own a defined type of resource in order to participate in blockchain activities. However, blockchains that use PoX are at risk of malicious users creating fake nodes on the network in order to claim consensus power. Usually, in this case, the longest chains in the network are considered to be the trusted chain. However, this does not prevent unfair power due to some nodes lacking the resources required to partake in blockchain decisions. Consensus algorithms that are Byzantine Fault Tolerant avoid this by having a pseudo-randomly selected leader determine if a decision should be made according to the decisions made by each node [21,22]. 

### 1.2. Blockchains in Healthcare

Although blockchains were initially used for cryptocurrencies and financial transactions, there are other possible uses for an unchangeable, trackable ledger. One major field where blockchains can make a positive impact is healthcare. Integrating blockchains with the electronic patient record systems can help solve some of the issues that exist in current healthcare infrastructures. For example, a patient may be given more control over who their data is transferred to. They will also be notified when another party, such as the doctor they refer to or the insurance company, attempts to share their data with someone else. Due to being notified, the patient will be able to decide whether or not they consent to their data being shared with others and can select who can view their medical information.

Another improvement that blockchains bring to healthcare is the ability to secure EMRs from unauthorized viewing and modification. With the way transactions occur in a blockchain, a user cannot view data unless they hold the required credentials to access it. This helps with patient privacy and security because only authorized parties will be able to access patients’ data while notifying them. Data integrity is preserved because the immutability of transactions and the order in which they are stored on the blockchain. The data is protected from modification unless the user is authorized and has permission to modify it. Because of this, patients will be able to be more confident about who views their personal and confidential health information. Depending on the implementation of the blockchains, patient may own the cryptographic keys for their data, and thus they will have the ability to select who on the blockchain is authorized to view their data.

The decentralized nature of blockchains ensures that patient data is not only safe from attacks that may cause downtime, but that it can also be recovered. In a traditional centralized environment, patient records would be stored on a database that is accessible from anywhere within a hospital. If the database is compromised or attacked, it may cripple an employee’s ability to access patient records. In the event that a malicious user decides to destroy data, EMRs may not be recoverable unless the files are backed up on another system. In a blockchain, data is distributed, and therefore exists on all nodes in the network, allowing it to be recoverable in the event that there is loss or corruption. Its decentralization means there is no single point of attack for adversaries to target. The owner of the data can also be confident that they will not need to update the data, since all updates to any data are broadcasted to all other nodes on the chain. 

**Figure 2:**
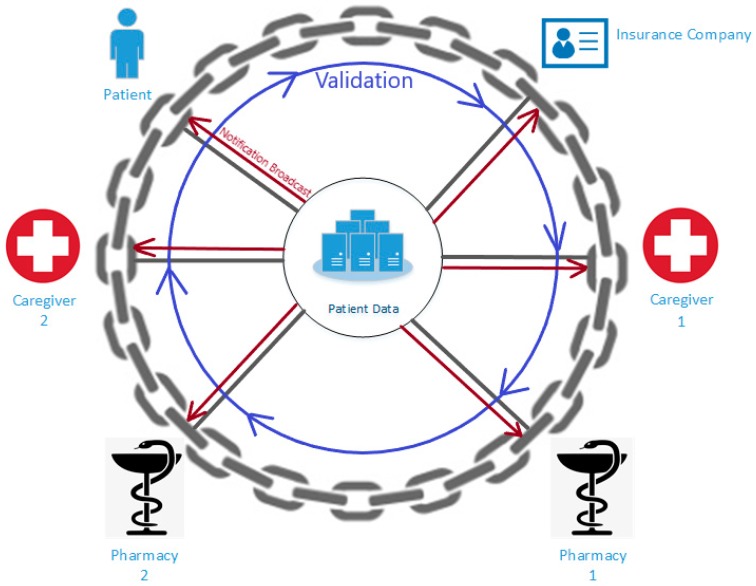
Healthcare blockchain conceptual model.

Blockchains also enable the ability to include other users and control their access. This can connect hospitals, insurance companies, and pharmacies together to improve provided services. An example of why this is beneficial for current healthcare infrastructures is the prescription of medications. When a doctor prescribes medicine to a patient, it can be viewed by authorized pharmacies and the insurance company. Pharmacists will be able to easily communicate with the associated insurance company to discover if the medication is covered by the patient’s insurance plan. Chaining these parties together reduces the paperwork and effort currently required to fill prescriptions for the patient to receive. Figure 2 depicts a conceptual model of how parties involved in healthcare can be networked through a blockchain. 

As can be seen from Figure 2, corresponding parties can be connected as part of a blockchain network. Each party represents a node on the chain. At the center of the chain, the assets, in this case, EMRs, are available to all nodes. When a node wishes to perform an action, such as accessing or modifying records, all nodes are informed of its occurrence through the notification broadcast. The other nodes must then validate the action through a consensus algorithm. If the majority of the nodes validate the action, a new block will be added to the chain, and another broadcast notification is sent.

### 1.3. Blockchains in Personlized Medicine

Along with the introduction of EHR/EMRs, personalized healthcare has been rising in popularity over the years as healthcare systems continue to evolve. The concept of personalized medicine revolves around categorizing patients according to certain common factors such as genomic data, race, age, or gender. For example, patients can be categorized based on genomic data collected through testing. Any health risks that a patient may have due to their gene variations may appear through testing, and caretakers can categorize patients according to that, as well. Treatments and health plans are given accordingly, depending on the characteristics of each category. Patient groups may also be targeted for pharmaceutical advertisements due to health concerns that may be common between group members. 

Although personalized medicine paves the way to further improve healthcare, especially eHealth, there are some issues that may arise from this model. The first major concern is the common issue of patient’s data privacy. For example, a patient’s data gathered through genomic testing, may also include family health risks. One issue related to this is the need to notify the patient’s family members of possible familial health risks, who may not consent to having their genome exposed, even if the patient gave the consent to be tested [23,24]. Another issue related to patient privacy is the possibility of a patient’s health data being needed for another study, which the patient may not consent to. These privacy issues hinder the patient’s confidence in the system and may deter them from seeking healthcare. The lack of confidence and fear of embarrassment or disclosure of private information may cause the patient to hesitate to provide accurate information or in some cases may give false information, which can influence treatment options, raise public health concerns, or even results in serious health complications and death [23,24].

Another concern is the availability of the health data collected from the patient. The labs that run the tests may store the results and raw data on their servers. However, if the data needs to be accessed by another party for any reason, it may be inaccessible [23]. Requesting access to the data and awaiting approval may take time and inconvenience both the caretaker making the request and the patient, who needs to give their consent. 

**Figure 3:**
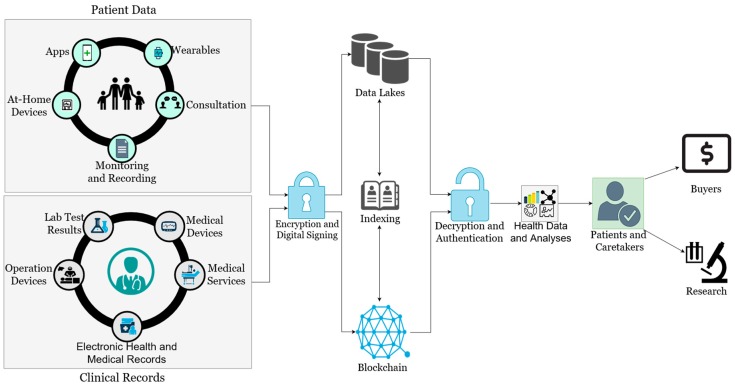
How blockchains can be integrated into personalized medicine.

Integrating blockchains into the personalized medicine model may address these issues. Encrypted transactions with only authorized, consented parties holding the keys to decrypt the data prevents others from viewing health data irrelevant to them. This adds some level of confidence in the healthcare system for the patient; knowing that their private information is only viewed by a select few. The notifications received whenever a transaction occurs, as well as the recording of all transactions, can also help the patient feel more at ease. Patients will be linked to their caretakers and any other stakeholders, allowing them to communicate anytime. Authorized doctors and researchers will also be able to access the data as needed, even if it is not directly available in their organization’s databases, as opposed to filing a request and waiting for it to be approved. This improved availability opens up the opportunity for patients to donate, and even sell, their health data to researchers for future patients and experiments. A summary of how blockchains can be integrated into personalized healthcare can be seen in Figure 3. 

Data generated by the patient and existing clinical records are encrypted and digitally signed before being stored into databases, or data lakes due to the immense size and amount of the data. Data from clinical trials can also be recorded and stored in the same manner, with researchers able to trace back through results to find patterns and correlations in data. When data is requested, it goes through authentication and decryption before disclosing the data to patients or caretakers. As can be seen from Figure 3, blockchains can be used as an index to link users to the actual location of the data being sought. Because a single patient’s file may be large due to the raw data coming from several sources, such as images or lab results, only links to the data should be communicated on the blockchain; the actual data will strictly be stored off-chain in the data lakes. An example of this can be seen with MedRec [25] and the Stony Brook Oncology project [26]. Transactions are time-stamped and recorded with the blockchain. With this model, patients can not only choose who will be able to access their data, but they can also choose to sell or directly hand their data to research facilities and pharmaceutical companies. In addition to this, pharmaceutical and insurance companies may also participate in the blockchain network for personalized medicine by requesting and utilizing the stored data to find potential participants for research projects. More details on this can be found in Reference [27].

### 1.4. Major Implementations 

There has been progress made with respect to implementing blockchains to improve healthcare infrastructures since the idea was first proposed in 2016. In 2016 there was a spike in the use of cryptocurrencies, and blockchains were viewed as a key component of financial transactions. Because of improved communication between parties when exchanging assets, researchers believed that blockchains could be used to solve problems found in current healthcare information systems. The immutable, distributed ledger has the capabilities to prevent unauthorized viewing, prevents unauthorized modification, improve access control, and notify all parties when access attempts or changes are made. 

The first major implementation of blockchains for healthcare systems was done by the Massachusetts Institute of Technology (MIT), whose researchers developed MedRec [25]. The purpose of this project was to improve the handling and exchanging of EMRs. The authors of the initial proposal sought to address four major issues: fragmented data, interoperability, patient-centricity, and research data. Three kinds of contracts were designed to handle data queries and establishing connections between the patient and the caretaker. However, issues remained regarding end-point security, scalability, and patient inference from connections within the blockchain [25]. In 2018, MedRec evolved into a usable system and addressed some of the issues that existed in the first iteration [28]. MedRec 2.0 addressed patient inference from transactions by introducing pseudonymity for communication and changed the way information is stored on the blockchain to address some scalability and privacy issues. However, patient inference still remains an issue from the metadata of their Ethereum address, as well as endpoint or provider database security [28].

Another implementation of blockchains in healthcare that is available for use is MedicalChain [11]. MedicalChain became available in 2018, with the aim of providing a private, secure, auditable, transparent, patient-centered system for caretakers and patients to communicate with each other. Similar to MedRec, it utilizes blockchains to assist in the management of EHRs. MedicalChain uses HyperLedger Fabric to power its system, due to the ability to provide access controls. This aids in the patient’s ability to control who access their medical records, while auditing and notifying any access and protecting their identity [11]. This also helps with MedicalChain’s functionality of providing a marketplace, which gives patients the opportunity to negotiate with third parties regarding the access or use of their data for purposes other than medical care, such as research. This is assisted by the currency provided by MedicalChain, known as MedTokens. However, in MedicalChain there are certain risks associated with the purchasing and storage of MedTokens, such as the possibility of tokens not being acquired by the patients altogether [11]. In addition, MedicalChain does not have any legal qualification for MedToken security, and thus patients and potential users should consider such risks when utilizing the provided services.

Patientory [29] has a similar model; using tokens to incentivize and offer services. This implementation of blockchains for the healthcare sector focused on the management of personal health data. Patientory focuses on patient privacy by strictly following HIPAA regulations, ensuring that all of their servers and processes are HIPAA compliant to protect the patient. Privacy and security are also ensured by the encryption of HIPAA-compliant servers, requiring the cryptographic to decrypt data [29]. During transmission, the data is re-encrypted using the requestor’s public key to prevent unauthorized parties from intercepting and viewing data. Similar to MedicalChain, Patientory also allows patients to share information with healthcare professionals, or even directly communicate with them through secure instant messaging. The tokens utilized by this platform can be used for purchasing additional storage space, or regulating smart contract functions during organization-to-organization communication. 

The authors of References [30,31] explored how blockchains can benefit the transfer of data in an Internet of Things (IoT) environment, with the latter focusing on pharmaceutical supply chains. The authors of Reference [31] also tested their work with a project called Modum.io AG, which was used to monitor shipment temperature and environmental status to meet the Good Distribution Practice (GDP) regulation set for medicinal product transportation. Smart electronic contracts are used to automatically gather data about the IoT network and notify parties of certain events. Those working in the IoT environment can have their mobile devices configured for this kind of setup. However, both authors have stated that latency can become an issue, especially in warehouses storing assets [31]. Privacy was also mentioned as an issue with using blockchains in an IoT environment, namely because of the ability to infer user identities based on the transactions that occur on the network, even if the traffic is encrypted [30,31]. Device activity can be monitored, and malicious users will be able to infer which parties perform certain activities, providing an opportunity for attack. 

As mentioned earlier in this paper, some researchers have proposed using cloud computing to improve on current healthcare systems. References [13], [32], and [33] have built on existing cloud-based healthcare infrastructures by integrating blockchains in order to improve upon the system. Reference [13] discussed an EHR/EMR system that already exists on a cloud and speculated if blockchains could be used to improve the security of patient records. There is more control over who has the ability to access and modify data since the majority of the authorized parties are required to agree on the intended actions. The authors of Reference [32] also sought to improve patient record security by integrating blockchains to include a security manager in the cloud to monitor transactions and ensure that parties have authenticated certificates when accessing the target data. In Reference [33], privacy in cloud-based healthcare systems is improved by introducing pseudonymity so that users will need a username and a password in order to access the blockchain platform, and smart electronic contracts for data management. The data being handled is also encrypted using what is known as Elliptic Curve Cryptography. This resulted in a project called MediBChain. However, even while building on a cloud-based eHealth system, there were challenges regarding the cost of implementation [32], system migration and interoperability [32,33], and satisfying privacy laws and regulations [13,32]. 

## 2. Materials and Methods

For this review, the fields of focus are healthcare and information systems security. Before deciding to proceed with the review and any projects that may follow, the following research questions are asked:

(1) How have blockchains been considered to improve healthcare information systems?
(2) What are the main areas of focus when implementing a blockchain for healthcare systems?
(3) What remaining issues still need to be addressed?
(4) Can blockchains be combined with artificial intelligence to further optimize personalized healthcare?

The review of the literature was done with texts found in the following databases:

(1) Google Scholar
(2) IEEE Access and Xplore
(3) Elsevier
(4) Springer

The query strings used to find the articles considered in this review included the terms ‘Blockchain(s)’, ‘Healthcare’, ‘eHealth’, ‘Medicine’, and ‘Personalized Medicine’. In order to reduce the number of results and ensure that the review is done with recent texts, the timeline of the search was limited to the past four years. Only articles published in English were considered for the review. The literature analyzed for the review typically consisted of models of how blockchains can be integrated into healthcare information systems, with a few theoretical texts and implementations. 

All of the texts about blockchains in healthcare that were selected for the review had to be relevant to topics related to:

(1) Electronic Health/Medical Records (EHR/EMR)
(2) Access Control
(3) Auditing
(4) Data/Record sharing

A publication was selected for review based on the abstract and its relevance to the research questions and the topics listed above. Once the search results were filtered out based on their abstracts, each publication was thoroughly read to ensure that it was indeed relevant to the subject at hand. If it was, the title and authors of the text were listed and categorized based on the ideas being proposed and what part of healthcare was focused on. 

Throughout this paper, a list of abbreviations and their definitions are used as per Table 1. 

## 3. Results

The articles included in this review were categorized based on the type of publication and the topics of focus. The types of publications considered are theoretical proposals, models, and other literature reviews. The two topics focused on are how blockchains can be integrated with eHealth in general to improve services of different fields, and how blockchains can be used to improve the handling of EHRs and EMRs. 

**Figure 4:**
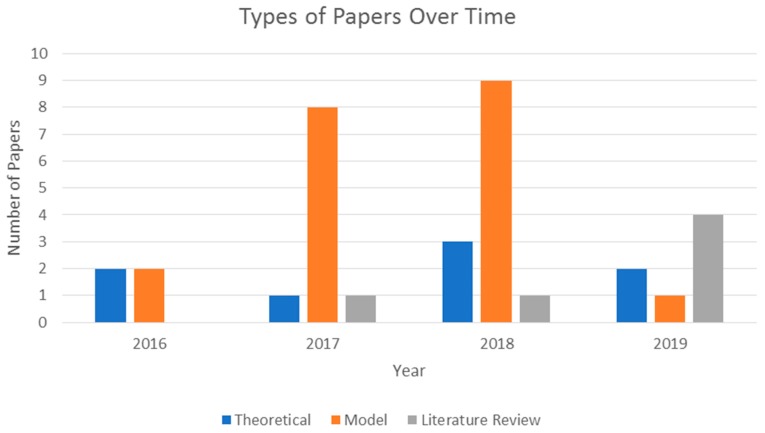
Types of papers published over time.

A total of 34 articles were selected for thorough review. With the exception of four publications, one of which being the initial paper introducing MIT’s MedRec, the reviewed papers selected for review were published after 2017. A summary of the types of papers published can be seen in Figure 4. 

**Figure 5:**
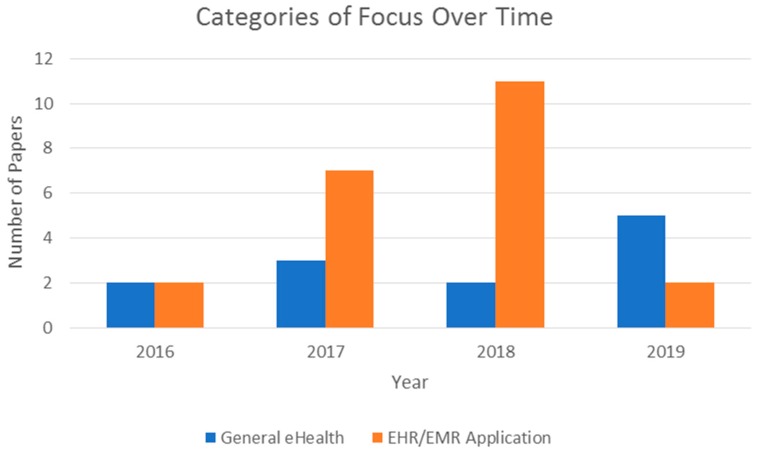
Comparison of focus between general eHealth topics and EHR/EMR applications over time.

As observed in Figure 4, the years 2017 and 2018 saw more model proposals related to the implementation of blockchains in healthcare infrastructure compared to the other types of papers. The figure also shows that there was an increase in literature reviews in 2019. This may be due to the number of models and theoretical papers published in 2017 and 2018; now that there have been some models and implementations done relevant to the subject at hand, there is an opportunity to review these works to determine existing issues and possible areas of focus for the future. One such area that was especially focused on is the use of blockchain technology to improve EHR and EMR services. Figure 5 depicts this trend among the reviewed publications. 

Based on the texts reviewed, the years 2017 and 2018 saw a significant increase in topics related to the possible use of blockchains for EHRs and EMRs. However, in 2019, so far, many papers reviewed how other topics can benefit from the features blockchains offer. This is likely due to the fact that, by now, there are several previously proposed models that have been implemented and published for use, such as MedRec [28] and MedicalChain [11].

**Figure 6:**
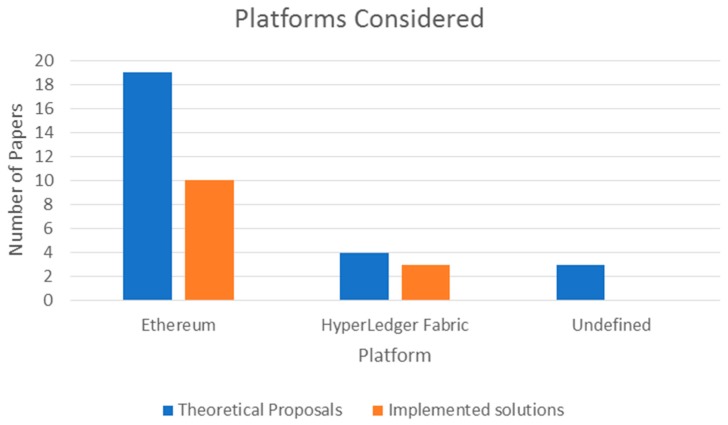
Blockchain platforms considered for solutions.

The publications reviewed mentioned the use of two possible platforms for their models or theory work: Ethereum or HyperLedger Fabric. Publications that were listed as literature reviews did not focus on a specific kind of platform and were not considered for this observation. The majority of models and theoretical proposals used Ethereum as their platform of choice, with 19 articles specifying the use or consideration of the platform. HyperLedger Fabric was discussed in four of the publications, whereas the remainder did not specify any preference regarding the platform used. Of the 13 projects, 10 used Ethereum, while the others used HyperLedger Fabric. A summary of this observation can be seen in Figure 6. 

The entire list of publications that were considered for this review can be seen in Table 2. 

Fourteen of the reviewed papers discussed projects that were proposed and tested with live data. Most of the projects specified the blockchain platform used during development, the implementation of a user interface, and the aim to comply with any privacy regulations regarding data privacy or transfer. A list of these projects can be found in Table 3. 

**Figure 7:**
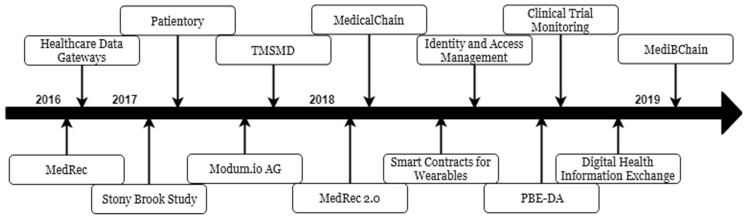
A timeline of the reviewed projects.

While reviewing the publications relevant to these projects, the benefits and disadvantages of each were noted down. The majority of the projects attempted to comply with the data privacy regulations specified by HIPAA. They also implemented pseudonymity in order to mask the identity of the patient during transactions within the chain. While the projects addressed privacy and security concerns, there were some technical concerns that remain to be addressed. For example, some of the projects faced an issue with system scalability; while testing, this may have not posed a problem. However, when implementing the proposed system for actual use, the number of users needs to be taken into consideration. A few projects also faced an issue with the need for cryptofuel in order to power consensus algorithms, such as that mentioned by MedicalChain [11] and MediBChain [33]. A timeline of these projects can be seen in Figure 7. Their advantages and disadvantages can be seen in Table 4. 

## 4. Discussion

After reviewing publications related to how blockchains can be used to improve current healthcare systems, the answers to the previously mentioned research questions can be pondered. The information gained from the review can help in the understanding of how this technology is being used in the field, as well as what challenges remain to be addressed. 

### 4.1. How Have Blockchains been Considered to Improve Healthcare Information Systems?

As seen from the reviewed texts, initial explorations for the use of blockchains in healthcare were to address issues with the existing EHR/EMR system. The first issue that was addressed by blockchains was the risky storage and sharing of EMRs. Currently, there are concerns over the ability to have unauthorized access and viewership of a patient’s information without their consent. This may be due to challenges in configuring access control for stored data [10,13,26,31,38,51,52], the lack of encryption of patient data [33], malicious internal users [13,26], or unsecured end-point devices [25,28,41,42,53]. Blockchains have been proposed either as a sole solution to concerns regarding EMR security or as a part of an existing solution, such as the cloud systems mentioned in References [13], [32], and [33]. 

In addition to securing patient data from unauthorized access, the implementation of blockchains for healthcare infrastructures improves the ability to audit access and modification attempts. Patients are notified whenever any other entity on the blockchain attempts to access their data. As the owners of their EMRs in the now patient-centric healthcare system, patients will be able to allow or deny any access or modification to their data, choose to share their data, and even whitelist parties for ease of access. 

Aside from improving EMR security, blockchains have also been used to manage pharmaceutical supply chains. Integrating blockchains and smart contracts into warehouse networks can allow the tracking of products, as well as their environmental status with the integration of IoT devices [30,31]. In relation to pharmaceuticals, blockchains have also been considered for detecting prescription fraud. References [9,50] have cited instances where blockchain technology was used to track prescriptions given to patients by tracking certain values once a prescription is filled. 

The use of blockchains with medical wearables was also explored. Some patients nowadays wear medical devices that allow caretakers to gather body status data from afar. Blockchains have been proposed to improve patient monitoring from a distance, due to its ability to automatically handle data. This further benefits both the patient and the caretaker because the data can be accessed in real time any time during the day [41,43,51,53]. The use of smart contracts with the blockchain can allow alerts to be sent to the caretakers involved once certain thresholds or events occur [41].

Lastly, there have been some improvements to the system architecture revolving communication and exchanging of data over the blockchain. The size of the data that healthcare professionals need to access, such as laboratory or scan results, may be large in size. Because of this, transferring data directly over the blockchain may be slow and even insecure. Several publications have suggested storing actual data files off-chain, while only communicating metadata and links to the required databases on-chain [25,28,37,38,45,47,49]. In doing so, transactions over the blockchain will be faster and more secure. This will also save space on the devices used to participate in the blockchain, since each node would have a copy of the blocks and assets. Storing data off-chain also provides an opportunity to improve access control configurations. 

### 4.2. What are the Main Areas of Focus When Implementing a Blockchain for Healthcare Systems?

When considering the integration of blockchains into modern day healthcare infrastructures, the first area of focus that researchers worked on was the improvement of EMR systems. Although EMRs are already a big improvement from physical files and paper records, the systems in use still contain privacy and security risks. The secure, immutable nature of blockchains address these concerns because only authorized parties will be able to view and modify patient records. Access control can also be added to separate read and write permissions. The presence of consensus algorithms and smart contracts give the patient control over which parties have the ability to view their data.

Another area of focus regarding the implementation of blockchains in healthcare is the detection of fraud attempts. Blockchains are considered true ledgers, meaning that the data that exists on the chain can be trusted. Any attempts to commit fraud, either insurance or prescription, are negated by the ability to view the transaction history. The immutability of blockchains allow organizations to keep track of any kind of information. Medical institutions, for example, can keep track of the degrees they hand out, as well as the achievements of their medical students [27]. This can help verify that the diplomas students claim to have are legitimate. The same idea can be applied to insurance procurement and pharmaceutical supply chain, where the prices of services and products, legal requirements, previous practices, and even tracking information for supplies can be recorded [27].

The integration of the Internet of Things with blockchains is another focus area. With the existence of medical wearables, such as heart rate or blood content monitors, caretakers can assess a patient without the need for frequent hospital visits. It is believed that blockchains can improve on this system by securing the communication of the data being gathered by body sensors worn by the patient. It also limits which parties have access to the gathered data and ensures that no data is lost because of its distribution to other nodes on the chain. The additional integration of smart electronic contracts can alert caretakers of certain events based on the live data being collected by the wearables. 

When considering the utilization of a blockchain to improve on healthcare systems, researchers generally focus on privacy and security. Patient records contain very sensitive personal information that malicious users can take advantage of. Even with traditional systems, patients are concerned about what parties their data is sent to and who has the ability to view their records. This may deter them from certain treatments, especially when a consent form regarding patient data is given to them for their signature. It is imperative that the privacy of patients and the security of their data is improved so that there is more confidence in healthcare systems. 

### 4.3. What Remaining Issues still Need to be Addressed?

As with any implementation, there are some issues that still need to be addressed while integrating blockchains into modern healthcare infrastructures. The first concern is the scalability of the system. Although the data to be stored on the chain can be controlled, the number of patients and parties partaking in the chain will continue to grow over time. At some point, computational resources, such as processing power and storage mediums, will become limited. This can hinder the services provided by the blockchain. Due to the continuous growth of the network, the scalability of the system and the amount of resources to power, this may become a concern. 

This leads to an issue regarding the cost of implementing such a system. Although blockchains can save money and resources in the long run due to automated moderation, the initial costs of installation may be high. Due to how consensus algorithms work, especially PoW, a large amount of processing power may be required. Because the blockchain networks in healthcare are expected to be large, implementing the required hardware and owning the appropriate devices to seamlessly utilize blockchain platforms may become expensive. 

Another issue that may arise is the concept of “garbage in, garbage out” (GIGO). This refers to a scenario related to user input, where a user may input incorrect or random data. The system will be forced to process this data, which may result in errors or a false output. The inclusion of blockchains in healthcare may face the same issue. Now that the patient is participating in their own healthcare, there is the possibility of “garbage” being input. The same possibility can occur with a caretaker or any other professional with modification or writing permissions. This may be accidental, due to computer illiteracy or misunderstanding the system, or intentional, in the event that the user inputting the data has malicious intentions. Although consensus algorithms would be present in the blockchain, GIGO is still possible if users of the blockchain do not pay attention to the data they are inputting into the system [9,41,47]. Issues can also arise if the blockchain network is implemented by IT professionals that are not familiar with blockchains and their configurations. This can lead to misguided diagnoses or wrong prescriptions, which may further lead to health complications or even the death of a patient in more serious situations. 

In any system or service, malicious users that seek to exploit loopholes to steal data or cause harm will always exist. Even if the blockchain is secure against attacks, end-point security remains an issue. If an end user has been compromised, the attacker may be able to corrupt the blockchain. They can infer information about the other users on the chain based on the communications that occur, steal data about the compromised victim, and give false information to be input on the chain. 

This leads to the final issue that will need to be addressed in order for blockchains to successfully improve healthcare systems. Data in a blockchain network is encrypted in such a way that only the owner of the data and consented parties that have the required key can access the data. In the event that these keys are lost or compromised, the owner will not be able to participate in the blockchain anymore. There needs to be a system that allows users to replace lost or stolen cryptographic keys, as well as informing other parties that may hold a shared key and updating them with the new key. 

### 4.4. Can Blockchains be Combined with Artificial Intelligence to Further Optimize Personalized Healthcare?

Data science is another field that has been seeking to improve the healthcare sector through the use of Artificial Intelligence (AI) and its machine learning algorithms. Similarly to blockchains, these decisions are made using special algorithms. However, where blockchains use collected data to attempt to protect data integrity, AI seeks to make predictions and informed decisions. A few examples of decisions to be made in the healthcare field are medical diagnoses, what kind of medications a patient may need, or required procedures.

As discussed previously in this paper, blockchains have been integrated with cloud computing and IoT to improve existing services and systems. Combining blockchains with AI can further improve several important parts of healthcare infrastructure, such as:

▪ Ensuring data integrity and validity
▪ Preventing and mitigating malicious activities
▪ Predictive analysis
▪ Real-time data analysis
▪ Managing data sharing

Improvements to data integrity and the prevention of traditional attacks have been addressed by blockchains systems. Large amounts of data, however, cannot be communicated directly through the blockchain without compromising system performance and some data security. Integrating AI algorithms into the system, however, will allow the data to be processed in advance so that only results and information are passed through the blockchain. Because the blockchain audits all transactions, healthcare professionals will still be able to understand how the data was processed and why informed decisions have been made. In order for decisions to be accurately made, the data processed to reach the conclusion will need to be reliable. Given the immutable, trusted nature of the data found on blockchains, the data set collected by machine learning algorithms would allow for proper decisions to be made. The availability of clean, accurate, reliable data eliminates the need for a data scientist to manually process data required for AI systems.

AI’s ability to make informed decisions may also assist with the mining of blocks, reducing the amount of computational resources required. Employing machine learning algorithms to mine blocks, as opposed to using traditional methods, can save both time and resources over time. For instance, if a health provider acquires suitable permissions to access the trusted and highly reliable patients’ data, then, through the use of AI, it will be possible to group patients based on molecular profiling, chemical reaction, gene variations, genetic disease, or any other profiling, which will be extremely helpful to advance personalized medicine.

## 5. Conclusions

The remaining security and privacy issues in current healthcare systems need to be addressed in order for patients to have more confidence in the medical professional and their respective clinics. One major area that needs to be focused on is EMR management. Digitalizing medical records has eased their storage and sharing. However, there remain issues around unauthorized access and disclosure, the centralized system that can be seen as a single point of attack, and patient medical information fragmentation in the event that multiple healthcare professionals are visited. 

In order to address these concerns, researchers are turning towards blockchains, a technology that was introduced when Bitcoin first came to light. Over time, there have been several developments to the original blockchain infrastructure so that it can be used for more than just cryptocurrencies and financial transactions. Researchers have begun looking into ways that blockchains can be used to improve current healthcare systems. Due to their immutable, transparent, distributed, and decentralized nature, blockchains can be used as a digital ledger to ease communication between patients, caretakers, and insurance companies. Patient data can also be stored in a single file, making it comprehensive and giving caretakers a better overview of the health history of the patient. 

A review of several published proposals for the inclusion of blockchains in healthcare has shown that the digital ledger technology can be used to improve current systems. Data is distributed and decentralized, preventing it from being lost and allowing it to be recovered in the event of an attack. Audit trails keep track of what transactions and modifications are made to patient records, while notifying all users on the network. Patients will be given more control over who has access to their data by selecting who carries the cryptographic keys required to decrypt and view it. 

However, the reviewed publications have also mentioned some areas of concern that will need to be addressed, such as issues with scalability. Healthcare systems are expected to handle large amounts of data for a large number of people, and blockchains will need to be able to grow in order to accommodate for such a number to provide seamless service. End-point security is also a concern, because although blockchains are secure, a single node being compromised may affect the entire chain. With the risk of a node being compromised, issues with key generation and replacement will also need to be addressed so that users can return to using the blockchain as soon as possible. 

Overall, the integration of blockchains into healthcare infrastructures shows great potential. Continuing research in this area will be beneficial to healthcare providers, patients, and other involved parties such as research institutions and insurance companies. Once the remaining issues with blockchains are overcome, healthcare systems can evolve for everyone’s benefit. 

## Figures and Tables

**Table 1:** Abbreviations and their definitions.

Abbreviation	Definition
EHR	Electronic Health Record
EMR	Electronic Medical Record
MIT	Massachusetts Institute of Technology
GUI	Graphical User Interface
App	Application
HIPAA	Health Insurance Portability and Accountability Act
GDP	Good Distribution Practice
GDPR	General Data Protection Regulation
TMSMD	Trusted Model for Sharing Medical Data
PoW	Proof-of-Work
PoS	Proof-of-Stake
GIGO	Garbage In, Garbage Out
AI	Artificial Intelligence

**Table 2:** List of publications reviewed.

Citation	Year of Publication	Implementation	Type	Category
[34]	2016	No	Theoretical	General eHealth
[30]	2016	No	Theoretical	General eHealth
[25]	2016	Yes	Model	EHR/EMR
[35]	2016	Yes	Model	EHR/EMR
[9]	2017	No	Literature Review	General eHealth
[31]	2017	Yes	Model	General eHealth
[10]	2017	No	Model	EHR/EMR
[36]	2017	No	Theoretical	EHR/EMR
[37]	2017	No	Model	General eHealth
[38]	2017	No	Model	Case Study—EHR/EMR
[26]	2017	Yes	Model	Case Study—EHR/EMR
[32]	2017	No	Model	EHR/EMR
[39]	2017	Yes	Model	EHR/EMR
[29]	2017	Yes	Model	EHR/EMR
[18]	2018	No	Literature Review	General eHealth
[40]	2018	No	Theoretical	General eHealth
[41]	2018	Yes	Model	EHR/EMR
[28]	2018	Yes	Model	EHR/EMR
[42]	2018	Yes	Model	EHR/EMR
[11]	2018	Yes	Model	EHR/EMR
[43]	2018	No	Theoretical	EHR/EMR
[13]	2018	No	Theoretical	EHR/EMR
[44]	2018	Yes	Model	EHR/EMR
[45]	2018	Yes	Model	EHR/EMR
[46]	2018	No	Model	EHR/EMR
[47]	2018	No	Model	EHR/EMR
[48]	2018	Yes	Model	EHR/EMR
[33]	2019	Yes	Model	EHR/EMR
[49]	2019	No	Literature Review	EHR/EMR
[50]	2019	No	Literature Review	General eHealth
[51]	2019	No	Literature Review	General eHealth
[52]	2019	No	Literature Review	General eHealth
[53]	2019	No	Theoretical	General eHealth
[54]	2019	No	Theoretical	General eHealth

**Table 3:** A list of tested projects.

Citation	Platform Used	User Interface	Regulation Compliance
[11]	HyperLedger Fabric	GUI & Mobile App	HIPAA & GDPR
[25]	Ethereum	Web Interface	HIPAA
[26]	HyperLedger Fabric	Unspecified	HIPAA
[28]	Ethereum	Web Interface	HIPAA
[29]	Ethereum	App	HIPAA
[31]	Ethereum	Android App	GDP ^1^
[33]	Ethereum	GUI	Unspecified
[35]	Ethereum	App	Unspecified
[39]	Ethereum	GUI	Unspecified
[41]	Ethereum	GUI	HIPAA
[42]	Ethereum	Unspecified	Unspecified
[44]	Ethereum	Unspecified	HIPAA
[45]	Ethereum	Web GUI	Unspecified
[48]	HyperLedger Fabric	App	Unspecified

^1^ Good Distribution Practice of medicinal products for human use, European Union.

**Table 4:** A summary of the reviewed projects.

Name of Project	Year	Advantages	Disadvantages
MedRec [25]	2016	First implemented project Addresses fragmented, slow accessibility, system interoperability, and Patient agency issues Encryption and audit trails HIPAA satisfaction	Data needs to be mined End-point security concerns Scalability issues
Healthcare Data Gateways [35]	2016	Data anonymization Simplifies singular patient data	Managing different kinds of data Keeping data private while running computations
Stony Brook Study [26]	2017	Patient has access control Data is encrypted Data is stored on the cloud Ensures service availability HIPAA satisfaction	Data is uncategorized Interoperability concerns with other systems and data types
Patientory [29]	2017	Real-time management Secure Storage Access Control	Pay for Storage Incentivizing Use of tokens
Modum.io AG [31]	2017	Use case with pharmaceutical management Ability to work offline Collects and tracks data from IoT devices Preserves data integrity	Forking Ethereum may cause failure DoS vulnerability Scalability issues
TMSMD ^1^ [39]	2017	Data and server encryption Patient record sharing Access Control	No privacy regulation compliance
MedRec 2.0 [28]	2018	Smart contracts to link addresses to data Pseudonymity No single point of attack Addresses smart contract vulnerability Restricts blockchain storage to identities HIPAA satisfaction	End-point security concerns Scalability Patient inference
MedicalChain [11]	2018	Allows communication between different clinicians Double encryption for more privacy and security Patient-centered	Requires cryptofuel/currency Chance of not acquiring tokens when buying them No control over tokens
Smart Contracts for Wearables [41]	2018	Data is stored on the cloud Majority of signatures needed for block validation Access control HIPAA satisfaction	Communication over open channel Transmission time of data Nodes need to remain online for consensus
Identity and Access Management [48]	2018	Access and identity control Registration for users Entities are patient-approved	Scalability issues
PBE-DA [42]	2018	IoT devices relieved from key generation and authentication Pseudonymity	Blockchain not fully integrated No mention of privacy regulation compliance Vulnerable to attack
Clinical Trials Monitoring [45]	2018	Encryption Access control Real-time monitoring	Permissions to access clinic databases required Interoperability concerns between different clinics and parties
Blockchain-based Digital Health Information Exchange [44]	2018	Data privacy Addresses policy and data access concerns Violation punishments	Scalability Data size
MediBChain [33]	2019	Only registered parties can participate Pseudonymity Raw data of other parties is inaccessible Authentication with activity	Smart contracts require cryptofuel No key theft/loss recovery No interoperability

^1^ Trusted Model for Sharing Medical Data.
